# *NOTCH2NLC* GGC repeats are not expanded in Italian amyotrophic lateral sclerosis patients

**DOI:** 10.1038/s41598-023-30393-6

**Published:** 2023-02-23

**Authors:** Arianna Manini, Delia Gagliardi, Megi Meneri, Sara Antognozzi, Roberto Del Bo, Giacomo Pietro Comi, Stefania Corti, Dario Ronchi

**Affiliations:** 1 Neuroscience Section, Department of Pathophysiology and Transplantation, Dino Ferrari Center, University of Milan, Milan, Italy; 2 Neurology Unit, Fondazione IRCCS Ca’ Granda Ospedale Maggiore Policlinico, Milan, Italy; 3 Neuromuscular and Rare Disease Unit, Fondazione IRCCS Ca’ Granda Ospedale Maggiore Policlinico, Milan, Italy

**Keywords:** Amyotrophic lateral sclerosis, Genetics, Neurology

## Abstract

Repeat expansions in genes other than *C9orf72* and *ATXN2* have been recently associated with Amyotrophic Lateral Sclerosis (ALS). Indeed, an abnormal number of GGC repeats in *NOTCH2NLC* has been recently reported in 0.7% of sporadic ALS patients from mainland China. This finding was not confirmed in an ALS cohort of subjects from Taiwan. As the involvement of expanded *NOTCH2NLC* alleles in ALS is debated, we addressed this point by evaluating *NOTCH2NLC* repeat expansions in an Italian cohort of ALS patients. A screening analysis of *NOTCH2NLC* GGC repeats was performed by repeat-primed polymerase chain reaction (RP-PCR) in a cohort of 385 probable/definite ALS Italian patients. Mean age at onset was 60.5 years (SD 13.7), and 60.9% were males. Sporadic cases were 357 (92.7%), and most patients had a spinal onset (71.8%). None of our patients showed the typical sawtooth tail pattern on RP-PCR, thus excluding abnormal repeat expansion in *NOTCH2NLC*. Overall, we suggest that *NOTCH2NLC* expanded alleles might be absent or at least extremely rare in ALS Italian patients. Further investigations in larger cohorts with different ethnic backgrounds are required to support the involvement of *NOTCH2NLC* in ALS.

## Introduction

The Notch 2 N-terminal like C gene (*NOTCH2NLC*), located at chromosome 1q21, differs from the other two human *NOTCH2* paralogs (*NOTCH2NLA* and *NOTCH2NLB*) for the presence of a repeat sequence (GGC)9(GGA)2(GGC)2 in the 5′ untranslated region (UTR), and for its enhanced expression in brain, especially in the prefrontal cortex^1,2^. Starting from 2019, *NOTCH2NLC* GGC repeat expansions in the 5′-UTR were found in patients affected by neuronal intranuclear inclusion disease (NIID), a neurodegenerative disorder characterized by eosinophilic, p62 and ubiquitin-positive intranuclear inclusions diffuse to different tissues, including the central and peripheral nervous systems^3–8^. NIID is a heterogeneous disorder characterized by a variety of neurological signs and symptoms, including cognitive impairment, parkinsonism, tremor, cerebellar ataxia, epilepsy, peripheral neuropathy, and autonomic dysfunction^2,5^. NIID is traditionally classified in three main types based on the predominant neurological features, namely muscle weakness-dominant, parkinsonism-dominant, and dementia-dominant^5^. *NOTCH2NLC* GGC repeat expansions have been reported in all these three forms, with a higher repeat size in the muscle weakness-dominant type^5^. An almost pathognomonic magnetic resonance imaging (MRI) marker of NIID is represented by a curvilinear hyperintensity at the corticomedullary junction at diffusion weighted imaging (DWI) sequences. However, its sensitivity is limited^2^.

By employing long-read sequencing (LRS), repeat-primed polymerase chain reaction (RP-PCR) and GC-rich PCR, the screening of *NOTCH2NLC* GGC repeat expansions has been rapidly extended to a variety of neurological disorders, including oculopharyngodistal myopathy (OPDM)^9,10^, Parkinson’s disease (PD)^11–16^, essential tremor (ET)^14,17–22^, multiple system atrophy (MSA)^14,23,24^, spinocerebellar ataxia (SCA)^5,14^, dementia [i.e., Alzheimer disease (AD), frontotemporal dementia (FTD), dementia with Lewy bodies (DLB), vascular dementia (VaD)]^5,25,26^, hereditary spastic paraplegia (HSP)^27^, peripheral neuropathy^5,28–30^, adult leukoencephalopathy^31–34^, and specifically cerebral small vessel disease^35^. However, the results of these studies have been spurious, so that the pathogenic role of *NOTCH2NLC* in neurological disorders beyond NIID is still debated.

In the last years, the discovery of a hexanucleotide repeat expansion in *chromosome 9 open reading frame 2* (*C9orf72*) as the main genetic cause of Amyotrophic Lateral Sclerosis (ALS) and the association between intermediate repeats in *ataxin 2* (*ATXN2*) with this disorder have suggested that repetitive sequences in human genome play a major role in ALS pathophysiology^36–38^. In this scenario, in 2020 Yuan and colleagues estimated the number of GGC repeats in the 5′-untranslated region (UTR) of *NOTCH2NLC* in 545 ALS patients from mainland China^39^. The authors found 4 ALS subjects carrying expanded alleles: two of them in the range of intermediate repeat numbers (44 and 54 repeats) and the others with pathogenic expansions of 96 and 143 GGC repeats. None of the age-matched 1305 controls displayed expanded alleles. Based on these data, the authors suggested that GGC repeat expansions in *NOTCH2NLC* might be also associated with ALS^3–5,17,31^. However, different authors failed to detect similar expanded alleles in *NOTCH2NLC* in other Chinese and Taiwanese ALS cohorts^5,40^.

In this scenario, we challenged the hypothesis that *NOTCH2NLC* GGC repeat expansions might be associated with ALS by evaluating their number in a cohort of Italian ALS patients.

## Results

**Figure 1:**
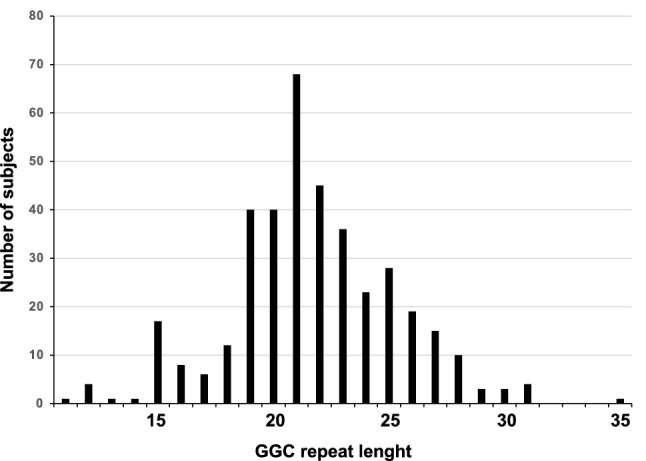
Distribution of the GGC repeat length of *NOTCH2NLC* in the 385 Italian patients with amyotrophic lateral sclerosis.

We enrolled 385 ALS patients, including 357 (92.7%) sporadic cases. Mean age at onset was 60.5 years (SD 13.7), and 60.9% were males. Most patients had a spinal onset (71.8%) rather than a bulbar one. The hexanucleotide repeat expansion in *C9orf72* and mutations in common ALS disease-causing genes [*superoxide dismutase 1* (*SOD1*), *TAR DNA-binding protein* (*TARDBP*) and *fused in sarcoma* (*FUS*)] were excluded. All patients were screened, and none of them showed the typical sawtooth tail pattern on RP-PCR, thus excluding the presence of abnormal expansions in the 5′-UTR of *NOTCH2NLC*. The estimated repeat sizes ranged from 11 to 35 in our cohort, as shown in Fig. 1. In Supplementary Fig. 1 we provide the pattern obtained by RP-PCR analysis in a representative negative control from our cohort compared to that of a known carrier of a *NOTCH2NLC* GGC repeat expansion (Supplementary Fig. 1)^14^. The number of patients enrolled, the ethnic background, the methods employed and the estimated *NOTCH2NLC* GGC repeat size were compared to previous studies performed both in ALS and in other neurological conditions, specifically HSP, NIID, OPDM, PD, ET, MSA, SCA, AD, FTD, DLB, VaD, peripheral neuropathy, adult leukoencephalopathy and specifically cerebral small vessel disease (Table 1).

**Table 1:** Studies describing *NOTCH2NLC* GGC repeat expansion screening in neurological disorders.

Disease References	Method	Origin	No of patients screened	No of carriers among patients (frequency)	*NOTCH2NLC* repeat size in patients (range)	No of controls screened	No of carriers among controls (frequency)	*NOTCH2NLC* repeat size in controls (range)	*NOTCH2NLC* repeat size in carriers (range)
ALS									
Tian et al.^5^	RP-PCR, GC-rich PCR	China	44 families	0	NA	211	0	5 to 38	NA
Yuan et al.^39^	RP-PCR, GC-rich PCR	China	545	4 (0.73%)	8 to 143	1305	0	4 to 41	44 to 143
Jih et al.^40^	RP-PCR	Taiwan	304	0	7 to 36	637	0	4 to 46	NA
Current study	RP-PCR	Italian	350	0	11 to 35	0	NA	NA	NA
HSP									
Hsu et al.^27^	RP-PCR	Taiwan	98	0	4 to 38	637	0	3 to 46	NA
NIID									
Sone et al.^3^	LRS, RP-PCR, GC-rich PCR	Japan	63	63 (100%)	71 to 183	545	1	− 6 to 61	61 to 183
Ishiura et al.^4^	TRhist, RP-PCR, SB	Japan and Malaysia	30	28 (93.3%)	7 to 180	1182	0	7 to 43	90 to 180
Tian et al.^5^	LRS, RP-PCR, GC-rich PCR	China	25 patients (4 families + 4 sporadic)	4 families + 4 sporadic (100%)	66 to 517	211	0	5 to 38	66 to 517
Deng et al.^6^	LRS, RP-PCR	China	15	15 (100%)	NA	0	NA	NA	NA
Chen et al.^7^	LRS, RP-PCR	China and Malaysia	12	12 (100%)	92 to 138	50	0	14 to 39	92 to 138
Yang et al.^8^	RP-PCR	China	602	10 (1.66%)	NA	0	NA	NA	95 to > 140
OPDM									
Ogasawara et al.^9^	RP-PCR, SB	Japan	211	7 (3.32%)	NA	0	NA	NA	> 100 to 674
Yu et al.^10^	LRS, RP-PCR	China	24	4 (16.67%)	NA	109	0	6 to 26	128 to 198
Parkinsonism									
Tian et al.^5^	RP-PCR, GC-rich PCR	China	205 families	3 families (1.5%)	NA	211	0	5 to 38	66 to 517
PD									
Ma et al.^12^	LRS, RP-PCR	Singapore	1000	13 (1.30%)	NA	1076	0	NA	41 to 130
Shi et al.^13^	RP-PCR, GC-rich PCR	China	1011	11 (1.09%)	NA	1134	0	6 to 39	41 to 52
Yau et al.^19^	RP-PCR	Europe	825	0	10 to 38	0	NA	NA	NA
Liu et al.^16^	RP-PCR, GC-rich PCR	China	1185	2 (0.17%)	NA	0	NA	NA	90 to 124
Billingsley et al.^15^	ExpansionHunter v4.02, LRS	Europe	6595	1 (0.02%)	NA	0	NA	NA	44 to 48
ET									
Sun et al.^17^	RP-PCR, GC-rich PCR	China	197	11 (5.58%)	7 to 138	1305	0	4 to 41	81 to 138
Liao et al.^18^	LRS	Europe	204	0	NA	406	0	NA	NA
Yau et al.^19^	RP-PCR	Europe	111	0	9 to 33	0	NA	NA	NA
Ng et al.^20^	RP-PCR, LRS	China	462	8 (1.73%)	NA	200	0	NA	47 to 107
Yan et al.^21^	RP-PCR, GC-rich PCR	China	228	3 (1.32%)	10 to 102	0	NA	NA	83 to 102
Zhou et al.^22^	RP-PCR, GC-rich PCR	China	597 families + 412 patients	27 families (4.52%) and 7 patients (1.70%)	NA	1085	0	4 to 41	41 to 250
Movements disorders (ET, PD, SCA, MSA)									
Yau et al.^14^	RP-PCR, SB, LRS	Europe	31,773	2 (< 0.01%)	NA	0	NA	NA	90 to 106
MSA									
Fang et al.^23^	RP-PCR, LRS	China	189	5 (2.65%)	NA	325	0	NA	101 to 266
Xu et al.^24^	RP-PCR	China	328	0	6 to 35	0	NA	NA	NA
SCA									
Tian et al.^5^	RP-PCR, GC-rich PCR	China	51 families	0	NA	211	0	5 to 38	NA
AD									
Tian et al.^5^	RP-PCR, GC-rich PCR	China	140 families	2 families (1.4%)	NA	211	0	5 to 38	66 to 517
Wu et al.^26^	RP-PCR, AL-PCR	China	39	1 (2.56%)	NA	0	NA	NA	43
Neurodegenerative dementias (AD, FTD, DLB, VaD)									
Jiao et al.^25^	RP-PCR, GC-rich PCR	China	1400	7 (0.50%)	NA	0	NA	NA	40 to 133
Neuropathy									
Tian et al.^5^	RP-PCR, GC-rich PCR	China	16 families	0	NA	211	0	5 to 38	NA
Wang et al.^28^	RP-PCR, AL-PCR	China	142	5 (3.52%)	6 to 206	100	0	6 to 26	126 to 206
Liao et al.^29^	RP-PCR, SB	Taiwan	127	7 (5.51%)	7 to 104	200	0	4 to 37	80 to 104
Wu et al.^30^	TP-PCR	China	128 (90 families)	2 families	NA	0	NA	NA	> 100
Adult leukoencephalopathy									
Okubo et al.^31^	RP-PCR, GC-rich PCR	Japan	93	12 (12.90%)	11 to > 89	58 (29 CBS and 29 PSP)	0	11 to 47	> 89
Yau et al.^33^	RP-PCR, GC-rich PCR	Europe	52	0	12–26	0	NA	NA	NA
Liu et al.^32^	RP-PCR, SB	Taiwan	163	34 (20.86%)	NA	0	NA	NA	73 to 323
Wu et al.^34^	RP-PCR, AL-PCR	China	41	39 (95.12%)	NA	0	NA	NA	87 to 159
Cerebral small vessel disease									
Wang et al.^35^	RP-PCR, AL-PCR	China	814	9 (1.11%)	NA	1134	0	6 to 39	41 to 98

*No* number, *MND* motor neuron disease, *ALS* amyotrophic lateral sclerosis, *HSP* hereditary spastic paraplegia, *NIID* neuronal intranuclear inclusion disease, *OPDM* oculopharyngodistal myopathy, *PD* Parkinson disease, *ET* essential tremor, *SCA* spinocerebellar ataxia, *MSA* multiple system atrophy, *AD* Alzheimer disease, *FTD* frontotemporal dementia, *DLB* dementia with Lewy bodies, *VaD* vascular dementia, *RP* repeat primed, *PCR* polymerase chain reaction, *LRS* long read, sequencing, *SB* Southern blot, *AL* amplicon length, *TP* triple primed, *CBS* corticobasal syndrome, *PSP* progressive supranuclear palsy, *NA* not available.

## Discussion

The four patients described by Yuan et al., who harbored GGC repeat expansions in *NOTCH2NLC*, showed similar clinical findings, including limb muscle weakness and atrophy, widespread fasciculations, dysarthria, dysphagia, dyspnea, and upper motor neuron signs^39^. The size of the abnormal repeat expansion was in the range of intermediate repeat numbers (between 43 and 59) in two cases, and in that of pathogenic expansions in the other two (96 and 143). Noteworthy, the two carriers of intermediate GGC repeat expansions died before reaching a definite diagnosis of ALS. High inter-individual clinical variability within families, nerve conduction abnormalities and intranuclear ubiquitin and p62-positive inclusions were identified in carriers of GGC repeat expansions in *NOTCH2NLC* and NIID-M patients. However, some significant differences argued against the presence of a unique clinical entity, including the significantly more severe phenotype and rapid deterioration of the four patients described by Yuan and colleagues, and the evidence of spontaneous activity on needle examination in more regions compared to NIID-M. Considering the different ranges of GGC repeat number detected in ALS (44–143) and NIID-M (118–517) patients, the authors suggested that the length of expansion might be related to the development of specific phenotypes, but further analysis are warranted to confirm this hypothesis. Alternatively, ALS with GGC repeat expansion in *NOTCH2NLC* might be a subtype of NIID previously undescribed.

To date, mutations in more than 30 genes have been associated with ALS. In Caucasian ALS patients the most recurring genetic defects are observed in *C9orf72* (familial ALS (fALS) 33.7%, sporadic ALS (sALS) 5.1%), followed by *SOD1* (fALS 14.8%, sALS 1.2%), *TARDBP* (fALS 4.2%, sALS 0.8%) and *FUS* (fALS 2.8%, sALS 0.3%)^41^. The proportion of mutated individuals might differ remarkably in different ethnic backgrounds, as previously observed for the mutation spectrum of ALS genes in the Chinese population^42^. However, a recent study failed to detect abnormal GGC repeats in *NOTCH2NLC* in a cohort of 304 unrelated Taiwan ALS patients, whereas an intermediate GGC repeat allele (46 repeats) was detected in 1 out of 637 control subjects^40^. Similarly, Tian and colleagues did not find expanded *NOTCH2NLC* alleles among 44 Chinese families affected by motor neuron disease, nor in 211 matched healthy controls^5^. While Tian et al. did not report the range of *NOTCH2NLC* repeats of their cohort, the one found by Jih and colleagues is in line the estimated repeat size of our ALS population (7–36 vs. 11–35)^5,40^.

Only six other works have performed *NOTCH2NLC* screening in patients of European descent, and specifically 2 in PD, 2 in ET, 1 in combined movement disorders, and 1 in adult leukoencephalopathy^11,14,15,19,33^. Out of a total of 38,820 European patients, only 2 were found to carry pathogenic *NOTCH2NLC* GGC repeat expansions (frequency 6.0 × 10^–5^). The first, who carried 118 *NOTCH2NLC* GGC repeats, was a Ukrainian woman affected by recurrent encephalopathy, whose skin biopsy revealed p62 and ubiquitin-positive inclusions in fibroblasts, endothelial cells, and serous glands. The second, instead, was an Italian man with postural tremor and a positive family history for tremor-dominant PD. The estimated number of GGC repeats in *NOTCH2NLC* was 90. Intriguingly, the employment of whole genome sequencing (WGS) revealed no significant differences in the repeat structure of the 5′-UTR of *NOTCH2NLC*, nor in its allelic frequency between individuals of European and East Asian descent^14^. This finding supports the hypothesis of a founder effect to explain the different distribution of NIID worldwide. Additionally, one PD patient was confirmed to carry an intermediate *NOTCH2NLC* GGC repeat expansions by LRS (estimated repeat size 44–48)^15^. Indeed, the recent development of cutting-edge techniques such as LRS has revolutionized our ability to detect long repetitive elements, copy number and structural variations, which cannot be revealed by conventional, short-read sequencing technologies. In this scenario, however, RP-PCR is still a valuable tool to confirm novel altered expansions of repeat units, or to perform screening in large cohorts.

This is the first assessment of the prevalence of GGC abnormal repeats in *NOTCH2NLC* in a European cohort of ALS patients. Although our study could be improved by the analysis of a control group and by the use of additional tests for a better detection of GGC repeat sizes or repeat interruptions, we think that these improvements are not expected to impact on the result.

Overall, we suggest that *NOTCH2NLC* expanded alleles might be absent or at least extremely rare in ALS Italian patients. Further investigations in larger cohorts with different ethnic backgrounds are required to support the involvement of *NOTCH2NLC* in ALS.

## Methods

The patients in our cohort meet the revised El Escorial criteria for probable or definite ALS^43^. Patients carrying a mutation in common ALS disease-causing genes (*C9orf72*, *SOD1*, *TARDBP* and *FUS*) were excluded. A screening analysis of GGC repeats in *NOTCH2NLC* was performed at the Fondazione IRCCS Ca’ Granda Ospedale Maggiore Policlinico by RP-PCR, as previously described^4^. Specifically, a slow-down PCR protocol was employed. After denaturation at 95 °C for 5 min, the cycling conditions were followed by: 50 cycles of 95 °C for 30 s, 98 °C for 10 s, 62 °C for 30 s and 72 °C for 2 min. We set the ramp rate to 95 °C and 72 °C to 2.5 °C s^−1^, and the one to 62 °C to 1.5 °C s^−1^. Electrophoresis was performed on a 3130 Genetic analyzer (Thermo Fisher Scientific, Waltham, MA) and the data were analyzed using GeneMapper software (Thermo Fisher Scientific). We performed RP-PCR also on a known positive control (patient B)^14^, as quality control assessment (Supplementary Fig. 1). All the probands provided written informed consent. The “Comitato Etico Milano Area 2 Fondazione IRCCS Ca’ Granda Ospedale Maggiore Policlinico” (Milan, Italy) approved the study. The study is in accordance with relevant guidelines and regulations.

## Supplementary Information

Supplementary Figure S1.

## Data Availability

The data that support the findings of this study are available on request from the corresponding author. The data are not publicly available due to privacy or ethical restrictions.
