# Supplementary material for: NOTCH2NLC GGC repeats are not expanded in Italian amyotrophic lateral sclerosis patients
Source: Sci Rep. 2023 Feb 23;13:3187. doi: 10.1038/s41598-023-30393-6 (PMC9950471; doi:10.1038/s41598-023-30393-6)

Supplementary Figure 1 – *NOTCH2NLC* RP-PCR analysis of a positive sample (Yau et al., Mov Disord. 2021;36:251-255. Patient B; the inset shows the magnification on the Y-axis in order to highlight the pattern of expanded peaks) and representative ALS patients from our cohort (all of them are negative for the *NOTCH2NLC* expansion).

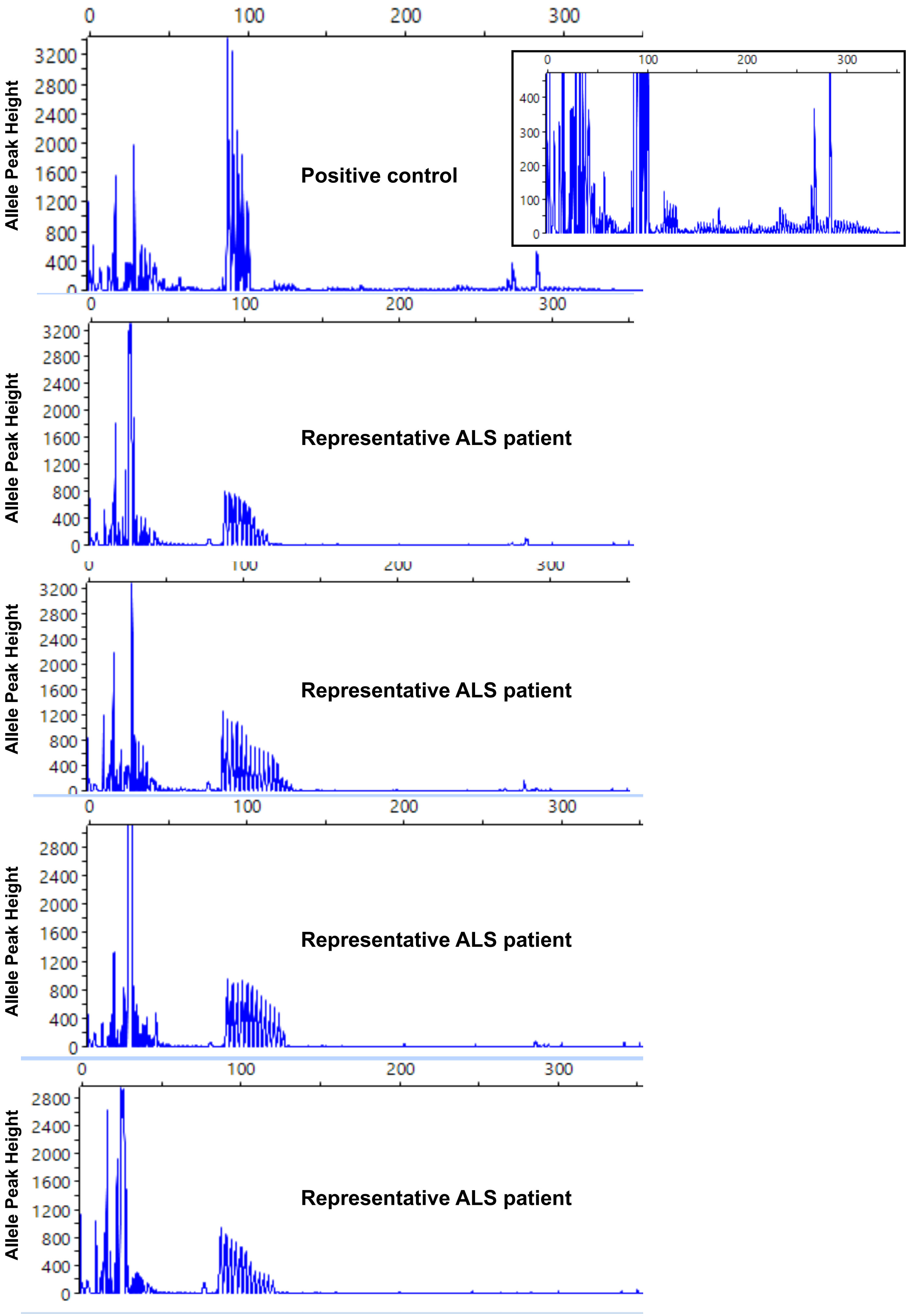

Supplement: Supplementary file 1 — Supplementary Figure S1. [file 41598_2023_30393_MOESM1_ESM.pdf]
